# Supplementary material for: Trichoderma longibrachiatum Inoculation Improves Drought Resistance and Growth of Pinus massoniana Seedlings through Regulating Physiological Responses and Soil Microbial Community
Source: J Fungi (Basel). 2023 Jun 21;9(7):694. doi: 10.3390/jof9070694 (PMC10381829; doi:10.3390/jof9070694)
Supplement: Supplementary file 1 [file jof-09-00694-s001.zip › jof-2424692-supplementary.pdf]

Journal of *Fungi*

MDPI

Supplementary

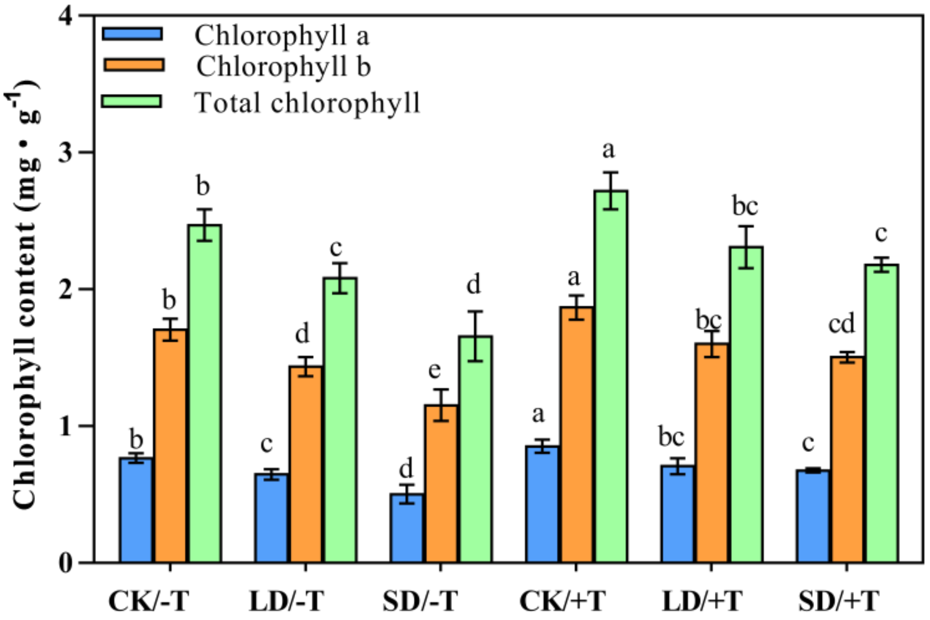

Figure S1. Measurement of chlorophyll content. Total chlorophyll, chlorophyll a and chlorophyll a were separately analyzed using one-way analysis of variance method. The different letters on the bars were statistically different at *P*<0.05.

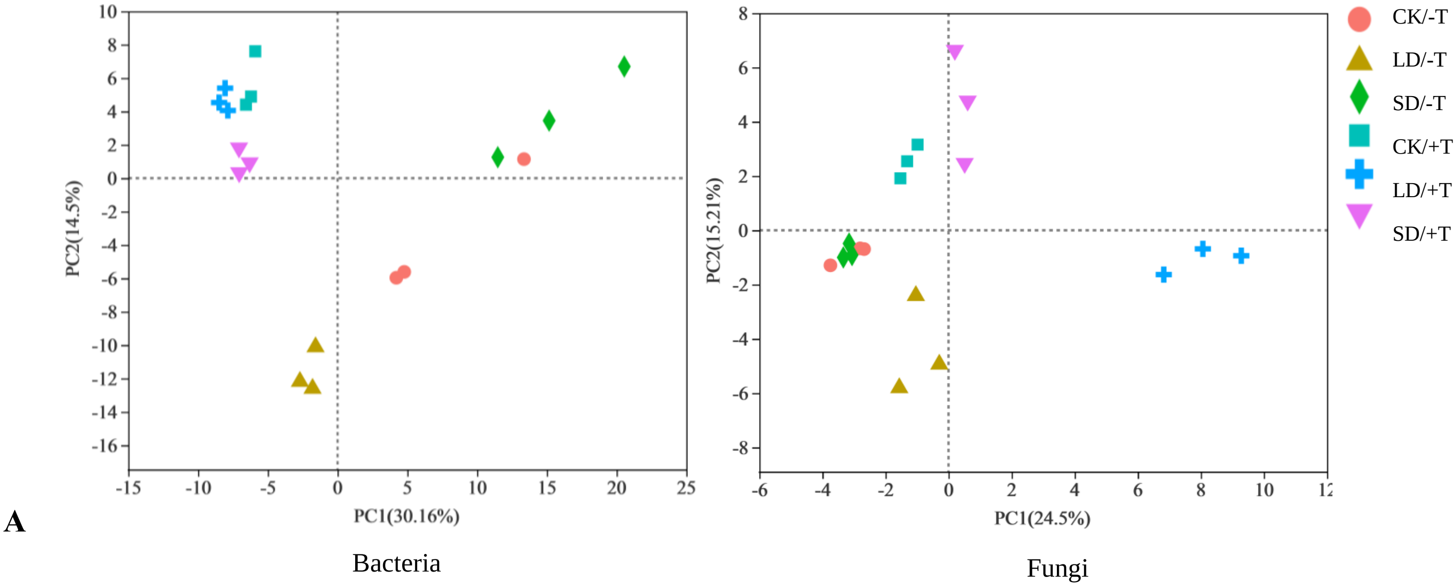

Figure S2. Principal component analysis (PCA) exhibited the compositions of bacterial (**A**) and fungal (**B**) communities in the different samples.

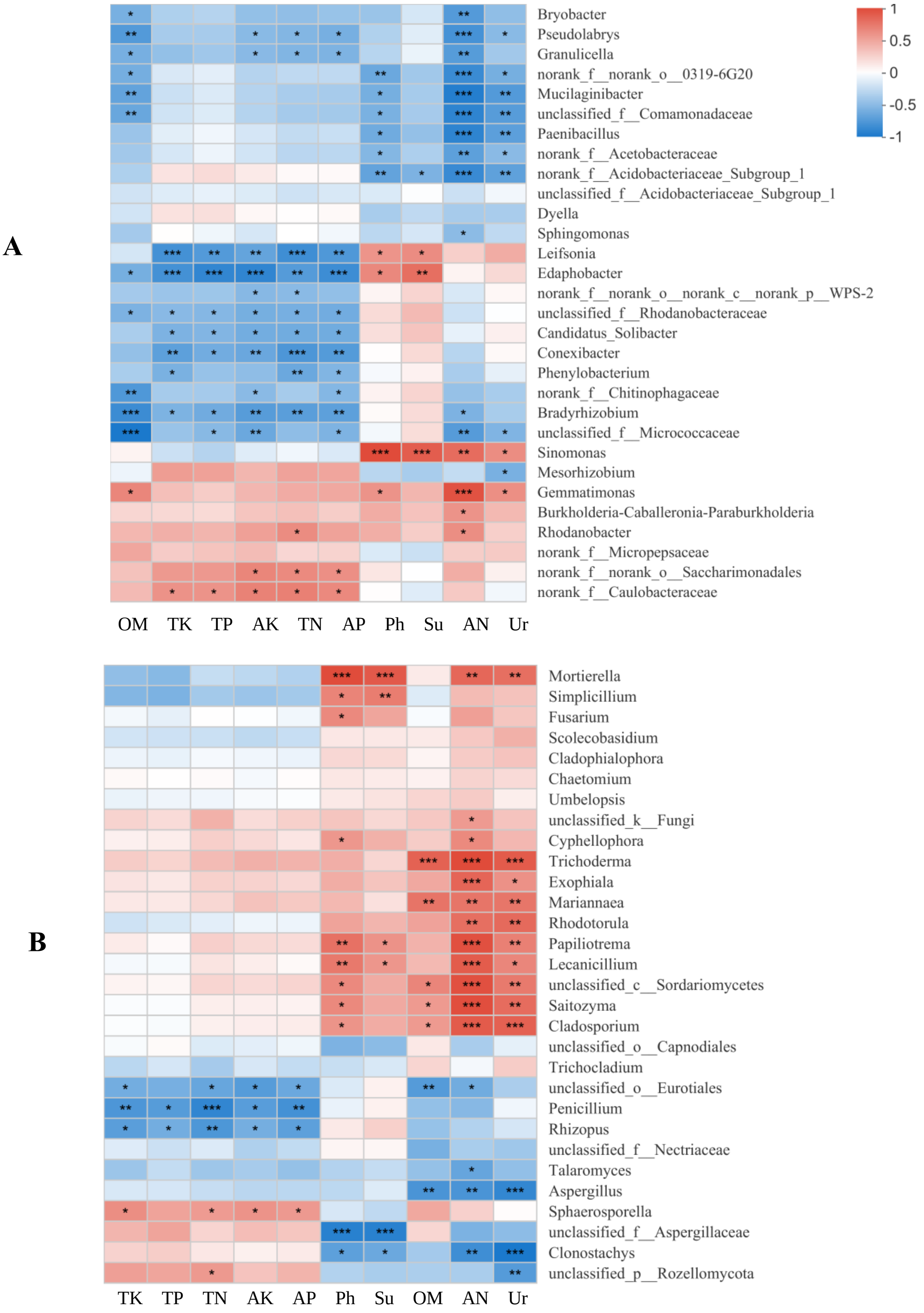

Figure S3. Spearman's correlation analysis. The correlation between soil physical-chemical properties and the top 30 genus in bacteria (**A**) and fungi (**B**). TN, total N; TK, total K; TP, total P; AP, available P; AN, available N; AK, available K; OM, organic matter; Su, Sucrase; Ph, Phphosphatase; Ur, urease. Significant levels were ****P*<0.001, ***P*<0.01, * *P*<0.05.

Table S1. Effects of *T. longibracteatum* on rhizosphere soil nutrients of *P. massoniana* seedlings under drought stress.

Treatments	TN ($g{\cdot}kg^{-1}$)	TK ($g{\cdot}kg^{-1}$)	TP ($g{\cdot}kg^{-1}$)	AP ($mg{\cdot}kg^{-1}$)	AK ($mg{\cdot}kg^{-1}$)	AN ($mg{\cdot}kg^{-1}$)	Organic <atter ($g{\cdot}kg^{-1}$)
CK/-T	0.60±0.04 b	17.35±0.26 b	1.04±0.03 b	19.09±0.74 b	330.38±7.38 b	71.17±2.02 e	6.04±0.19 c

LD/-T	0.37±0.04 c	13.48±0.41 d	0.66±0.04 d	14.48±0.55 c	318.11±3.25 c	106.17±2.02 c	6.39±0.21 bc
SD/-T	0.25±0.04 d	10.15±0.59 e	0.24±0.03 f	10.44±0.52 d	265.33±1.47 e	89.83±3.50 d	5.32±0.18 d
CK/+T	0.84±0.07 a	21.90±0.53 a	1.50±0.05 a	29.01±0.83 a	368.28±2.06 a	110.83±4.04 c	6.56±0.28 b
LD/+T	0.56±0.07 b	16.21±0.35 c	0.84±0.06 c	19.27±0.22 b	333.58±0.44 b	169.17±5.35 a	7.11±0.30 a
SD/+T	0.39±0.04 c	13.35±0.07 d	0.48±0.04 e	14.05±0.41 c	297.45±2.74 d	122.50±0.01 b	5.95±0.36 c

Table S2. Effects of *T. longibracteatum* on soil enzyme activities in rhizosphere roil. of *P. massoniana* seedlings under drought stress

Treatments	Sucrase ($mg \cdot g^{-1} \cdot d^{-1}$)	Phosphatase ($mg \cdot g^{-1} \cdot d^{-1}$)	Urease ($mg \cdot g^{-1} \cdot d^{-1}$)
CK/-T	7.77±0.28 e	58.77±1.50 d	7.67±0.89 d
LD/-T	11.68±0.04 c	70.48±2.15 c	14.17±0.66 b
SD/-T	14.75±0.10 b	76.03±3.31 b	10.49±1.35 c
CK/+T	10.28±0.12 d	68.49±1.91 c	10.65±2.21 c
LD/+T	14.58±0.17 b	81.39±3.78 b	18.98±0.97 a
SD/+T	18.42±0.04 a	90.52±0.91 a	14.27±0.43 b

Table S3. The Spearman's correlation analysis of soil physicochemical properties.

Soil physicochemical properties	OM	TN	TK	TP	AN	AK	AP	Su	Ph	Ur
OM (Organic matter, $g \cdot kg^{-1}$)	1									
TN (Total N, $g \cdot kg^{-1}$)	0.54*	1								
TK (Total K, $g \cdot kg^{-1}$)	0.53*	0.94**	1							
TP (Total P, $g \cdot kg^{-1}$)	0.56*	0.93**	0.96**	1						
AN (Available N, $mg \cdot kg^{-1}$)	0.62**	0.10	0.02	-0.04	1					
AK (Available K, $mg \cdot kg^{-1}$)	0.69**	0.89**	0.91**	0.95**	0.13	1				
AP (Available P, $mg \cdot kg^{-1}$)	0.65**	0.96**	0.96**	0.95**	0.14	0.93**	1			
Su (Sucrase, $mg \cdot g^{-1} \cdot d^{-1}$)	0.50*	-0.16	-0.25	-0.29	0.87**	-0.08	-0.09	1		
Ph (Phphosphatase, $mg \cdot g^{-1} \cdot d^{-1}$)	-0.03	-0.49*	-0.60**	-0.65**	0.69**	-0.49*	-0.50*	0.68**	1	
Ur (Urease, $mg \cdot g^{-1} \cdot d^{-1}$)	-0.26	-0.63**	-0.75**	-0.80**	0.52*	-0.67**	-0.66**	0.60**	0.96**	1

Significant levels were **P<0.01, *P<0.05.

Table S4. Alpha diversity index of the microbial community under different treatments.

	Fungi				Bacteria			
Treatments	shannon	simpson	ace	chao	shannon	simpson	ace	chao
CK/-T	1.81±0.16 c	0.28±0.04 ab	105.58±5.78 c	104.90±7.14 b	4.29±0.18 ab	0.038±0.0049 c	485.45±10.62 a	490.93±15.44 a
LD/-T	1.86±0.21 c	0.28±0.06 ab	104.40±8.05 c	105.43±7.58 b	4.15±0.09 bc	0.035±0.0018 c	421.04±25.68 b	424.00±34.48 b
SD/-T	1.79±0.16 c	0.32±0.04 a	92.90±1.90 d	92.23±1.64 c	4.44±0.11 a	0.030±0.0040 c	475.52±7.51 a	484.55±16.03 a
CK/+T	2.18±0.28 b	0.19±0.04 bc	128.89±2.20 ab	131.90±6.40 a	3.96±0.15 c	0.060±0.012 b	477.51±4.52 a	481.69±4.04 a
LD/+T	1.61±0.08 c	0.32±0.04 a	120.08±5.24 b	121.22±8.71 a	3.48±0.21 d	0.096±0.015 a	416.70±10.66 b	416.23±11.54 b

SD/+T	2.70±0.08 a	0.11±0.01 c	130.41±6.03 a	132.56±7.30 a	4.01±0.12 c	0.047±0.0075 bc	437.27±14.62 b	437.53±13.24 b
